# Exploring the Implications of Golgi Apparatus Dysfunction in Bone Diseases

**DOI:** 10.7759/cureus.56982

**Published:** 2024-03-26

**Authors:** Georgian L Iacobescu, Antonio-Daniel Corlatescu, Mihnea Popa, Loredana Iacobescu, Catalin Cirstoiu, Carmen Orban

**Affiliations:** 1 Orthopaedics and Traumatology Department, University Emergency Hospital, Bucharest, ROU; 2 Orthopaedics and Traumatology, Carol Davila University of Medicine and Pharmacy, Bucharest, ROU; 3 General Medicine, Carol Davila University of Medicine and Pharmacy, Bucharest, ROU; 4 Cardiology, Carol Davila University of Medicine and Pharmacy, Bucharest, ROU; 5 Cardiology Department, University Emergency Hospital, Bucharest, ROU; 6 Anaesthesiology and Critical Care, Carol Davila University of Medicine and Pharmacy, Bucharest, ROU; 7 Anaesthesiology and Critical Care Department, University Emergency Hospital, Bucharest, ROU

**Keywords:** signaling pathways, glycosylation, vesicular trafficking, protein sorting, bone diseases, golgi apparatus

## Abstract

The Golgi apparatus is an organelle responsible for protein processing, sorting, and transport in cells. Recent research has shed light on its possible role in the pathogenesis of various bone diseases. This review seeks to explore its significance in osteoporosis, osteogenesis imperfecta, and other bone conditions such as dysplasias. Numerous lines of evidence demonstrate that perturbations to Golgi apparatus function can disrupt post-translational protein modification, folding and trafficking functions crucial for bone formation, mineralization, and remodeling. Abnormalities related to glycosylation, protein sorting, or vesicular transport in Golgi have been associated with altered osteoblast and osteoclast function, compromised extracellular matrix composition, as well as disrupted signaling pathways involved with homeostasis of bones. Mutations or dysregulation of Golgi-associated proteins, including golgins and coat protein complex I and coat protein complex II coat components, have also been implicated in bone diseases. Such genetic alterations may disrupt Golgi structure, membrane dynamics, and protein transport, leading to bone phenotype abnormalities. Understanding the links between Golgi apparatus dysfunction and bone diseases could provide novel insights into disease pathogenesis and potential therapeutic targets. Future research should focus on unraveling specific molecular mechanisms underlying Golgi dysfunction associated with bone diseases to develop targeted interventions for restoring normal bone homeostasis while decreasing clinical manifestations associated with these issues.

## Introduction and background

The Golgi complex, also referred to as the Golgi apparatus or body, occupies an integral position among essential organelles present in eukaryotic cells. It plays an integral role in key cellular processes such as intracellular transport, protein processing, and secretion. Its discovery over a century ago was an enormous milestone in cell biology, revolutionizing our understanding of cell organization and function [1].

Beginning in the late 19th century, Italian physician and scientist Camillo Golgi began unraveling its secrets. With hard work and innovative staining techniques that provided remarkable views into cell functioning, Camillo Golgi developed tools that enabled him to gain insights into this intricate organelle’s inner workings. Camillo Golgi made an astounding discovery while studying nerve cells using his groundbreaking staining method known as the Golgi staining technique or black reaction in 1898. While studying them he noticed an intricate network of interconnected membrane-bound sacs interspersed among their cytoplasm; later becoming known as the Golgi apparatus and thus forever immortalizing Golgi in scientific history [1].

First referred to as an internal reticular apparatus or Golgi’s internal reticular apparatus, its true significance and functionality gradually unfolded over the following decades. George Palade made strides in the 1950s using advanced electron microscopy techniques to explore the complex structural composition of the Golgi apparatus while highlighting its vital roles in various cell processes [2].

The discovery of the Golgi complex shed light on cellular architecture and function organization within cells, prompting further investigations of its significance both physiologically and pathologically. Scientists have since unraveled many functions performed by this multifaceted structure, including protein modification, packaging sorting lipid metabolism signaling pathways.

**Figure 1:**
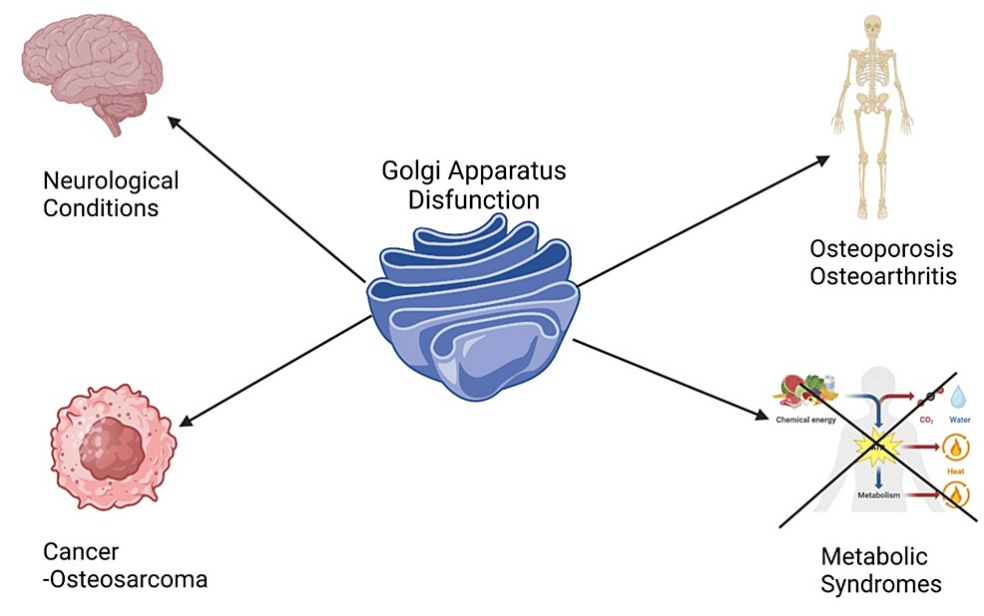
Golgi apparatus dysfunction-associated disorders. These malfunctions arise from abnormal functioning or structural alterations of the Golgi apparatus, an organelle responsible for protein modification, sorting, and trafficking within cells. The “X” on the Metabolic Syndromes pathway represents the altering of a normal metabolism. This is an original figure created by the authors.

Today, the Golgi complex plays a central role within cells, orchestrating molecules as they move along its stacked cisternae and vesicular transport systems. Beyond basic cell biology, its relevance extends further. Its malfunction has been implicated in numerous human diseases spanning neurological conditions, such as neurodegenerative diseases, metabolic syndromes, and cancer [3,4]. Neurodegenerative diseases have been associated with changes to Golgi structure and function that could contribute to disease progression or be indicative of specific pathological features. Such modifications could alter disease progression or feature as specific pathologies [5] (Figure 1).

Golgi apparatus dysfunctions

The Golgi apparatus, found in nearly all eukaryotic cells, consists of stacks of cisternae. Within each of these cisternae are specific enzymes responsible for the glycosylation of proteins and lipids as they traverse along their path through this apparatus in an ordered fashion. While its exact mechanisms remain under discussion, evidence is in favor of cisternal maturation being its likely mechanism [6]. According to this model, cargo molecules are held within cisternae that undergo maturation as they move from the cis to the trans region of the Golgi stack. This maturation process is assisted by recycling Golgi enzymes via retrograde vesicles that bud from later cisternae’s rims and fuse with earlier ones; another possible mechanism could involve either anterograde trafficking within the Golgi stack itself or tubular connections among different cisternae or both approaches could occur simultaneously [7]. An important role in bone-related disease regarding the Golgi apparatus functions is represented by the process of glycosylation. Glycosylation is a complex enzymatic process that adds sugar molecules to proteins, lipids, and other molecules, significantly altering their structure and function. Glycosylation plays an integral role in skeletal development by contributing to bone matrix formation, mineralization process control, and regulation of signaling pathways that manage growth within the skeleton system [8,9].

The Golgi apparatus plays an integral role in cell disruption when its proteins, including structural, trafficking, glycosylation enzymes, and ion channels, experience functional defects. Traditionally, this organelle regulates protein secretion from endoplasmic reticulum (ER) into Golgi. Recent studies have demonstrated that many Golgi proteins participate in unconventional protein secretion pathways (UPSs) which bypass the Golgi. These UPSs become activated during periods of stress, protein degradation, autophagy, or energy metabolism. As pathways associated with diseases are being linked with Golgi proteins, these have recently been investigated as potential therapeutic targets. This review highlights their significance in disease pathogenesis while exploring novel therapies that target them as possible treatments.

Numerous diseases have been linked with altered Golgi morphology and function. Abnormalities associated with Golgi abnormalities include fragmentation of its apparatus and impaired trafficking from ER to Golgi. Ribbon degradation during mitosis or differentiation occurs naturally while pathological conditions such as apoptosis or neurodegenerative diseases commonly exhibit fragmented Gol-gis fragmentation. Dysfunction of stress response mechanisms, protein homeostasis mechanisms, protein trafficking, or glycosylation has been implicated as being contributors to disease pathogenesis. We will explore these links further as we discuss disease pathogenesis through Golgi abnormalities [10].

Protein glycosylation

Protein glycosylation occurs in two distinct ways depending on its location within a cell. Within the cytosol and nucleus, proteins undergo O-GlcNAcylation in which one or more sugar molecules called b-N-acetylglucosamine (GlcNAc) are attached to serine or threonine residues for O-GlcNAcylation. This process has profound ramifications on protein interactions, stability, and activity as well as regulation of essential processes such as transcription, metabolism apoptosis organelle biogenesis, and transport [11,12].

ER and Golgi apparatus, however, present unique forms of glycosylation. Secretory and transmembrane proteins undergo modification with oligosaccharides called glycans that bind specifically to amino acid side chains of secretory and transmembrane proteins. Lumenal glycosylation can further be divided into four distinct categories according to where their sugar chains attach. An example of each category based on protein attachment sites is presented below.

N-glycosylation involves attaching glycans to the amide group of asparagine residues. O-glycosylation refers to linking glycans with the hydroxyl groups on serine, threonine, hydroxylysine, or tyrosine residues.

C-mannosylation involves attaching a mannose molecule directly to the C2 atom of tryptophan through a C-C bond, while glypiation refers to linking proteins with glycosylphosphatidylinositol anchors in membranes through glycan linkers.

N-glycosylation stands out among these forms of glycosylation as being particularly well-researched and studied.

N-glycans, important sugar chains attached to proteins, undergo a unique synthesis process. At first, they are produced as precursors known as lipid-linked oligosaccharides (LLOs). Each LLO contains 14 sugar chains arranged in an L-shape. An enzyme known as oligosaccharyltransferase then transfers these chains onto newly synthesized proteins during protein translation from their lumen of ER location during translation from precursors LLOs [13].

Before proteins reach the Golgi apparatus for transport to plasma membranes, three glucose and one mannose residues are removed from their sugar chains in the ER to produce high-mannose-type sugar chains similar to those seen in lower eukaryotes. Unfortunately in differentiated vertebrate cell types, these high-mannose N-glycans rarely reach cell surfaces due to extensive modifications during transport via the Golgi apparatus to plasma membranes [14,15].

In the cis-Golgi, high-mannose N-glycans from the ER are further modified. By attaching GlcNAc sugar molecules to mannose residues, sugar branches are created. As N-glycans continue their journey through the Golgi region, they undergo further modifications such as galactose (Gal), sialic acid, and fucose decoration in late Golgi (also called trans-Golgi). Such modifications give rise to complex N-glycan structures with various structures and functions. Lumenal O-glycosylation differs significantly from N-glycosylation in that its process is much more varied and complex, yet its exact mechanism remains unknown. Higher eukaryotic cells typically produce two primary O-glycan types, namely, shorter mucin-type glycans and longer glycosaminoglycan chains attached to proteoglycans that are synthesized within the Golgi apparatus for greater diversity among their cell’s structures [16,17].

Congenital disorders of glycosylation

Congenital disorders of glycosylation (CDGs) encompass a spectrum of genetic conditions that can result in various skeletal abnormalities, including craniofacial dysmorphisms, limb anomalies, joint contractures, and vertebral anomalies. These manifestations have a devastating impact on the quality of life and functional abilities of those affected by CDGs. By understanding specific CDG skeletal features associated with each condition, it becomes easier to identify and treat them early.

Studies have provided insights into the link between CDGs and compromised bone mineral density (BMD), which increases the risk for osteoporosis and fractures and CDGs. Mechanisms behind decreased BMD in CDGs may include disruptions in the glycosylation of matrix proteins; changes to calcium metabolism pathways; impaired function of osteoblast and osteoclast cells responsible for bone formation/resorption, respectively; or dysregulation of key factors that regulate bone health. By uncovering these underlying mechanisms, researchers can gain a better understanding of the intricate relationship between glycosylation processes and skeletal development, leading to targeted interventions and management strategies aimed at mitigating any associated skeletal complications from CDGs. A greater comprehension of their connection could improve diagnosis, treatment, and overall care provided for individuals affected by CDGs [18].

Golgi apparatus and genetics

From a genetic perspective, according to Kobayashi et al. [19], EXT1 and EXT2, the gene products associated with hereditary multiple exostoses (HME), have been found to localize to the Golgi apparatus. HME is an uncommon genetic condition characterized by multiple benign bone tumors called osteochondromas. Mutations of *EXT1 *and *EXT2 *genes have long been recognized as primary contributors. Both genes encode glycosyltransferases that play an essential role in producing heparan sulfate, an essential constituent of extracellular matrix composition. It has been demonstrated that EXT1 and EXT2 proteins interact to form a complex within the Golgi apparatus that facilitates heparan sulfate synthesis. Their localization in Golgi also suggests they play an integral role in the glycosylation of proteins intended for secretion or membrane localization, perhaps through glycosylation processes [19].

**Figure 2:**
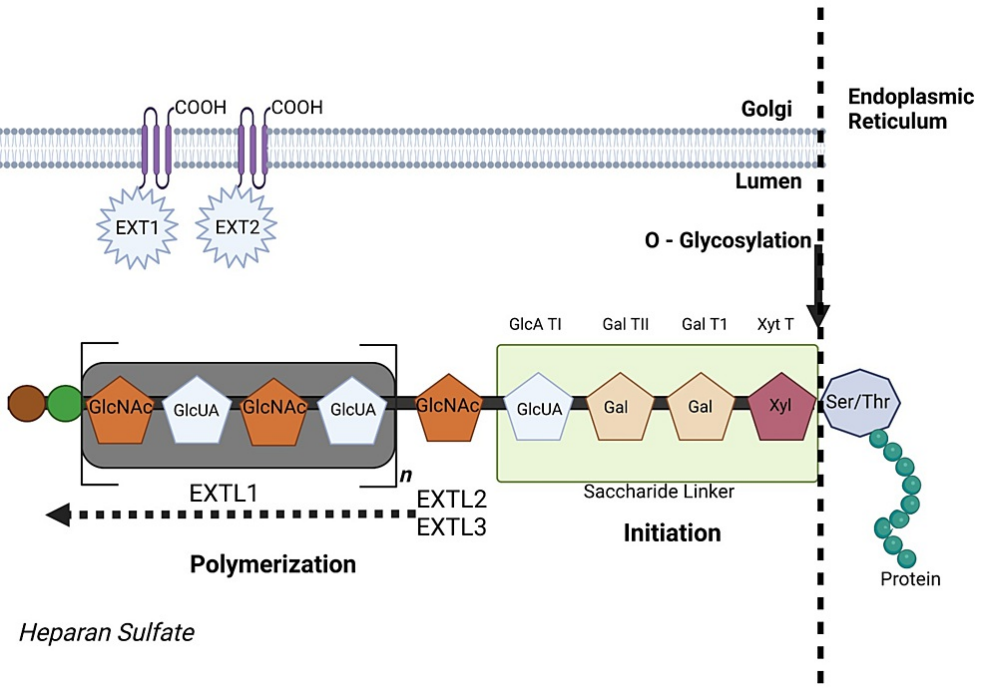
Heparan sulfate (HS) biosynthesis. HS synthesis begins with a tetrasaccharide on a core protein to make HSPG: xylose, two galactoses, and glucuronic acid. EXTL2’s enzyme initiates the chain with N-acetylglucosamine. Exostosin-1 and 2 then alternately extend it with glucuronic acid and N-acetylglucosamine, producing full HS proteoglycans. EXT1 and EXT2: EXT1 stands for exostosin glycosyltransferase 1 and EXT2 for exostosin glycosyltransferase 2. EXTL1, EXTL2, and EXTL3: EXTL stands for exostosin-like, and these genes also encode enzymes involved in HS chain elongation. EXTL1 is exostosin-like 1, EXTL2 is exostosin-like 2, and EXTL3 is exostosin-like 3. GlcNAc: N-acetylglucosamine, which is a sugar molecule that serves as a building block for polymers such as HS. GlcUA: Glucuronic acid, another sugar component of HS. Gal: Galactose, a type of sugar molecule. Xyl: Xylose, a sugar molecule that links to serine or threonine residues on proteins during glycosylation. Ser/Thr: These represent the amino acids serine (Ser) and threonine (Thr), which are the sites on proteins where O-glycosylation can occur. GlcA TI, Gal TI, and Gal TII: These are likely abbreviations for specific glycosyltransferases that add glucuronic acid (GlcA) and galactose (Gal) residues during the glycosylation process. TI and TII: Type I and type II isoenzymes of these glycosyltransferases. Xyt T: This might stand for xylosyltransferase, the enzyme that adds a xylose molecule to serine or threonine residues on proteins, starting the glycosylation process. The “n” in the image represents the polymerization process, indicating that the sugars are added repeatedly to create a long chain. This is an original figure created by the authors.

Disruption of EXT1 and EXT2 function results in disruptions in heparan sulfate synthesis, with potentially disastrous ramifications on signaling pathways involved with cell growth, development, and skeletal morphogenesis, contributing to osteochondromas observed among those suffering from HME. Further research must be conducted to fully comprehend how EXT1 and EXT2 act in the Golgi apparatus and influence heparan sulfate production and its pathogenesis to develop effective treatment protocols against this disorder (Figure 2).

Other Golgi complex dysfunctions that have clear effects on the skeletal systems are caused by disrupted vesicular trafficking and abnormal protein secretion at the osteoblast and osteoclast levels. The Golgi apparatus serves as an important hub for vesicular trafficking, transporting proteins from ER to various cell components, including plasma membrane and subcellular compartments. If this process becomes impaired, it could result in impaired protein transport and result in abnormal cell functions or subcellular compartmentalization. However, when the Golgi function is compromised, it can compromise normal vesicle formation, transport, and fusion processes, ultimately leading to altered protein secretion and intracellular accumulation. Bone cells often suffer from dysfunction that restricts their release of essential components of bone matrix, growth factors, and signaling molecules necessary for tissue homeostasis and maintenance of activity. Such disruptions have serious repercussions for activity levels as well as overall tissue equilibrium [20].

When a failure of the Golgi complex, regarding protein secretion and transportation, occurs, osteoblasts and osteoclasts may produce aberrant protein secretion patterns. Osteoblasts secrete essential bone matrix proteins such as collagen, osteocalcin, and osteopontin into their respective matrix layers, while osteoclasts release enzymes involved in bone resorption. When their Golgi apparatus fails, normal secretion processes of these proteins are disrupted, which impacts bone health as well as remodeling processes overall. This impairment can have severe implications on health as well as remodeling processes overall, potentially having detrimental impacts both on health as well as remodeling efforts. Regulated bone remodeling is crucial to the well-being and strength of bone tissue. This process involves the coordinated activity of two key cell types, i.e., osteoclasts for bone resorption, and osteoblasts involved in formation, in achieving balanced remodeling of bones. Achieving balanced remodeling requires coordination among these cells. Synaptotagmin VII stands out as an intriguing factor in this regulation process, playing an essential role in modulating osteoclast and osteoblast secretions of signaling molecules such as growth factors and cytokines that contribute to communication, recruitment, and differentiation processes within bone cells, ultimately impacting overall bone remodeling processes. The deficiencies or malfunctions can result in abnormal bone remodeling, leading to conditions such as osteoporosis or excessive bone resorption. Due to its dysfunction, osteoclast and osteoblast secretions become out of balance, jeopardizing both the integrity and strength of bone tissue. Synaptotagmin VII plays an essential role in controlling osteoclast and osteoblast secretions of factors that regulate bone remodeling processes, leading to imbalanced remodeling patterns and compromised integrity in conditions such as osteoporosis and excessive bone resorption. Deficits or malfunction of this protein could contribute to imbalanced remodeling patterns leading to compromised integrity in bone tissues such as osteoporosis or excessive bone resorption [21,22].

## Review

Osteoporosis and Golgi apparatus dysfunction

Osteoporosis, an increasingly prevalent bone disorder characterized by diminished bone density and increased fracture susceptibility, is affected by various factors, including genetic and cellular mechanisms. Recent studies have shed light on an intriguing correlation between Golgi apparatus dysfunction and osteoporosis development. The Golgi apparatus, an essential organelle responsible for protein processing and transport, plays a crucial role in bone cell function and tissue integrity. Disruptions to Golgi-mediated processes involving vesicular trafficking, protein sorting, and post-translational modifications can contribute to the development or progression of osteoporosis [3].

Osteoporosis is an increasing global health challenge that affects more than 200 million individuals globally. Unfortunately, due to its silent nature, it often goes undiagnosed or undertreated until complications such as fractures occur and require medical intervention [23]. Raising awareness and understanding of osteoporosis is crucial to timely recognition and proper treatment. Comprehensive treatment involves various therapies and interventions, including calcium and vitamin D supplementation, bisphosphonates, estrogen therapy with selective estrogen receptor modulators, calcitonin, parathyroid hormone, and balance and exercise training programs, as well as minimally invasive spine procedures such as vertebroplasty or kyphoplasty. This multidisciplinary strategy takes an integrated approach to meeting the complex needs of osteoporosis patients. Encouragingly, there has been evidence of increasing medical intervention as healthcare providers become aware of its significance and proactive treatment measures [24]. In addition, osteoblastic cells play an important role in developing the bone matrix, and it is worth mentioning that they indirectly affect calcium homeostasis [25] which is involved in the apparition of osteoporosis, calcium being essential to maintaining bone density. Sufficient levels of calcium are necessary for the mineralization of bone tissue, and thus essential in maintaining strength and avoiding fractures. Osteoporosis results from an imbalance between bone resorption by osteoclasts breaking down existing tissue and formation by osteoblasts building new bone, leading to a net loss of calcium from bones over time, progressive decreases in density, as well as an increase in fracture risk. Hormonal changes such as menopause, insufficient calcium intake, vitamin D deficiency, and the natural aging process can disrupt calcium homeostasis and contribute to osteoporosis development [26,27].

GORAB, also known as SCYL1BP1, is an integral member of the Golgin family that can be found localized to the trans side of the Golgi apparatus. This protein plays an essential role in maintaining integrity and function within this complex organelle. Mutations in the *GORAB* gene have been associated with gerodermia osteodysplastica, an extremely rare genetic disorder with symptoms including wrinkled skin and osteoporosis in individuals living with it [28].

GORAB is essential in coat protein complex (COP)I trafficking, or protein transport within the Golgi apparatus. As a scaffolding factor for COPI assembly at trans-Golgi networks (TGNs), it acts as an anchor between Scyl1 and COPI assembly at TGNs. Mutations in *GORAB* inhibit this assembly, leading to impaired recycling of trans-Golgi enzymes and improper glycosylation of proteins [29,30].

GMAP-210, also known as TRIP11, is another example of the impact of Golgi matrix protein loss on living systems. GMAP-210 is an integral Golgin protein found in cis-Golgi networks (CGNs), where it contributes to asymmetric membrane tethering. Animal studies have demonstrated how mutations in *TRIP11 *led to the loss of GMAP-210, which, in turn, caused abnormal Golgi-mediated glycosylation and transport of proteins between cells within both chondrocytes and osteoblasts of mice chondrocytes and osteoblasts. *GMAP-210* mutations have also been identified among patients suffering from severe conditions known as chondrodysplasia achondrogenesis 1A, an extreme disorder characterized by abnormal bone development.

These examples demonstrate the key role Golgi matrix proteins such as GORAB and GMAP-210 play in maintaining proper Golgi function, protein trafficking, and glycosylation processes. If these processes become disrupted, they can have serious repercussions for bone cell functions and lead to disorders such as osteoporosis or chondrodysplasia. Understanding their mechanisms could shed light on developing targeted therapeutic approaches for these conditions [31].

Loss of *COPB2* gene function has been identified as the cause of a rare genetic disorder characterized by coatopathy, osteoporosis, and developmental delay. COPB2 plays a crucial role in producing COPII which transports proteins between ER and the Golgi apparatus.

**Figure 3:**
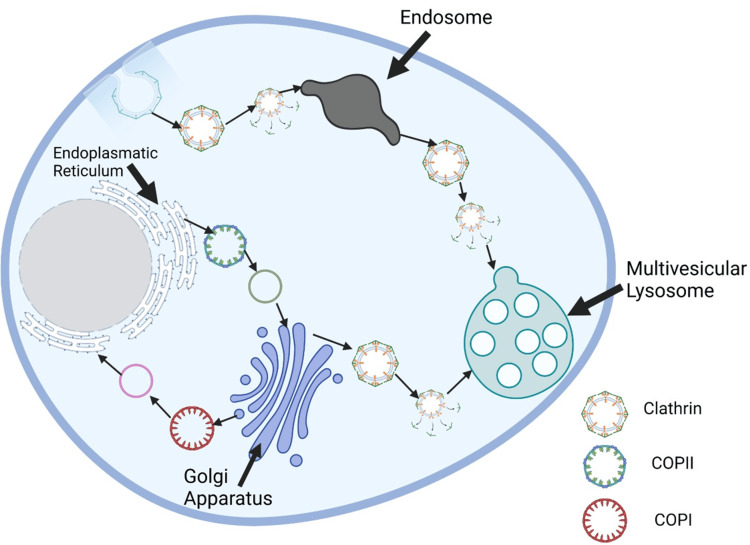
The evolution of three types of intracellular transport vesicles. The evolution of three types of intracellular transport vesicles involved with intracellular transport: coat protein complex I, coat protein complex II, and Clathrin-coated vesicles. This visual depiction emphasizes their complexity and interconnectivity. This is an original figure created by the authors.

Individuals carrying *COPB2 *mutations exhibit various clinical manifestations, such as osteoporosis, which weakens bones and increases fracture risk, as well as developmental delay or impaired nervous systems. Disruption of COPII-mediated protein transport results in abnormal secretion and distribution of proteins within cells, leading to dysfunction and impacting various biological processes. For bone health specifically, defective transport prevents proper synthesis and secretion of essential components such as bone matrix proteins, growth factors, and signaling molecules which play key roles in bone development and maintenance (Figure 3) [32,33].

Golgi apparatus involvement in osteoarthritis

The Golgi apparatus, with its role in protein processing and transport, has emerged as an important player in osteoarthritis, a degenerative joint condition marked by cartilage breakdown and inflammation. One key role of the Golgi apparatus in osteoarthritis is its involvement in the production and secretion of essential extracellular matrix components, including collagen and proteoglycans, that are crucial for cartilage health. Dysfunctions in Golgi-mediated processes, such as protein glycosylation or transport, may lead to abnormal matrix synthesis that compromises its quality and structure. The Golgi apparatus plays an essential role in cellular stress responses and redox balance, both known to contribute to osteoarthritis pathogenesis. If its function becomes impaired, it may cause increased oxidative stress and damage within joint tissues, resulting in increased oxidative stress and tissue destruction. It plays a pivotal role in the release of pro-inflammatory cytokines and enzymes which contribute to osteoarthritis symptoms, while dysregulated Golgi secretion may contribute to imbalanced levels of these inflammatory mediators, further exacerbating joint tissue damage [34,35].

Mutations in the *Matrilin-3* gene have emerged as key contributors to osteoarthritis, a degenerative joint condition. Matrilin-3 is an extracellular matrix protein essential in maintaining the structure and function of cartilage. Scientific investigations have identified specific mutations within *Matrilin-3* that cause changes to its structure and function. This causes disruptions to the normal assembly and organization of its extracellular matrix which compromises its ability to withstand mechanical stress while keeping joints functioning at an optimum level. At present, researchers are still trying to understand how *Matrilin-3* mutations contribute to osteoarthritis; however, it is thought that these mutations could hinder interactions between *Matrilin-3* and other components of cartilage, disrupt signaling pathways involved with maintaining homeostasis, and potentially increase cartilage degradation and inflammation. *Matrilin-3* mutations have been linked with early-onset osteoarthritis, primarily affecting joints in the hands, knees, and spine. Individuals carrying such mutations may experience faster degeneration of cartilage degradation as well as an increased risk for osteoarthritis at a younger age [36].

Understanding the role of *Matrilin-3* mutations in osteoarthritis will allow us to identify at-risk individuals and develop targeted interventions against this debilitating condition [36,37].

Another relevant osteoarthritis-related gene is the *COG5 *gene which is an integral part of the conserved oligomeric Golgi (COG) complex, which plays an essential role in maintaining Golgi apparatus structure and function. Mutations or disruptions within this complex, such as with the *COG5 *gene itself, may result in impaired processes such as vesicular trafficking, protein glycosylation, or Golgi morphology, potentially disrupting several cellular functions, including cartilage formation chondrogenesis or osteogenesis. These abnormalities have far-reaching cellular implications affecting cartilage formation or osteogenesis.

Notably, the *COG5 *gene from the C7q22 region associated with osteoarthritis susceptibility has been extensively researched for its involvement in glycosylation defects, chondrogenesis and osteogenesis, Wnt signaling, as well as inhibition of *COG5 *gene expression. Through experiments that involve such inhibition, it has been seen that it leads to glycosylation defects affecting proteins crucial for chondrogenesis and osteogenesis, creating disruptions in protein glycosylation processes with long-term consequences on cartilage development and functionality of cartilage and bone tissues [38,39].

Golgi complex and bone cancer

Osteosarcoma is one of the most frequently diagnosed primary bone cancers among children and adolescents and carries an increased risk for both local and distant metastasis. Although progress has been made over recent decades, osteosarcoma does not show significant improvements in overall prognosis. Furthermore, molecular mechanisms involved with osteosarcoma metastasis remain poorly understood. Little attention had been paid previously to how glycans play an integral role in its development or progression, sparking our interest further to research how changes affect metastatic behavior within cancer cells with differing metastatic properties and analyze how these impact its malignant properties.

ST6GAL1 is an enzyme that plays an essential role in adding terminal galactose and sialic acid glucans to N-glycans, as part of protein processing and trafficking pathways. The Golgi compartment plays an essential role in protein processing and trafficking pathways. Disruption of this pathway has been implicated in various diseases, including cancer and hypoxia, yet its exact mechanisms remain elusive. Further studies are required to elucidate how ST6GAL1 fits within this complex network of glycosyltransferases within the Golgi apparatus [40]. Sialyltransferases are enzymes responsible for moving sialic acid from activated CMP-NeuAc to sialylated glycolipids or N/O-linked sugar chains of glycoproteins. We compared the expression profiles of sialyltransferase genes between MG-63 and Saos-2 cells using real-time polymerase chain reaction array analysis, finding ST6Gal-I showed the greatest variance. Based on this finding, we hypothesize that ST6Gal-I may play an essential role in promoting the malignant progression of osteosarcoma cells [41].

Recently, there has been growing recognition of the role that long non-coding RNAs (lncRNAs) play in establishing and progressing tumors. LncRNAs feature complex secondary and tertiary structures and have the potential to regulate gene expression through interactions with DNA, RNA, and proteins [42,43]. These lncRNAs serve the following four key roles: signaling (acting as indicators of transcriptional activity), guiding (recruiting chromatin-modifying enzymes to specific genes for remodeling or epigenetic regulation), decoying (serving as “miRNA sponges” to sequester miRNAs that target transcription factors or protein factors away from chromatin to the nucleus), and scaffolding (mediating stability of ribonucleoprotein complexes). Recent reports indicate lncRNA involvement in the regulation of GOLPH3 as well [44].

GOLPH3 is a protein that serves as an effector of phosphatidylinositol-4-phosphate, playing an integral role in maintaining the structure and anterograde trafficking of Golgi apparatuses by connecting trans-Golgi membranes with F-actin through interactions with myosin 18A (MYO18A). Furthermore, this gene has also been identified as being amplified in various cancerous conditions in human bodies. GOLPH3 and MYO18A work together to form a complex that exerts a pulling force on the trans-Golgi membrane, which plays a role in the budding of vesicles for anterograde trafficking. Not only does this force facilitate vesicle formation but it also alters the Golgi apparatus morphology by stretching its ribbon around the nucleus and flattening its cisternae, giving its characteristic appearance when observed through fluorescence or electron microscopy. Furthermore, through screening, it has also been found that GOLPH3 also serves as an oncogene, often amplified during various human cancers [45].

As such, we determined five lncRNAs associated with both GOLPH3 and miR-142-5p to assess their significance using expression analysis and survival analysis using The Cancer Genome Atlas data in lung adenocarcinoma patients; among these, TUG1 was identified as the key lncRNA. TUG1 is a recently identified oncogenic lncRNA that exhibits abnormal upregulation across various cancer types. Notably, TUG1 was significantly overexpressed in colorectal cancer patients and served as an oncogene in osteosarcoma by competitively binding with miR-335-5p [44,46].

Table 1 summarizes the Golgi complex mechanisms and proteins involved in osteoarticular diseases.

**Table 1:** A summary of Golgi complex and osteoarticular diseases. [28,31,32,36,38,45]. SCYL1BP1: SCY1-like 1-binding protein 1 (also known as Golgi-associated plant pathogenesis-related protein 1); GMAP-210: Golgi microtubule-associated protein 210; COPB2: COPI coat complex subunit beta 2; COG5: component of oligomeric Golgi complex 5; GOLPH3: Golgi phosphoprotein 3; MYO18A: myosin XVIIIA

Disease	Golgi mechanisms	Genes and Golgi proteins involved
Osteoporosis	Protein glycosylation, calcium homeostasis, and secretion of extracellular matrix proteins	SCYL1BP1 protein, GMAP-210 protein, and *COPB2 *gene
Osteoarthritis	Protein processing and secretion, cellular signaling, and inflammation	Matrilin-3 protein and COG5 protein
Osteosarcoma	Protein secretion, glycosylation, and intracellular trafficking	GOLPH3 protein and MYO18A protein

## Conclusions

The Golgi apparatus has emerged as an essential factor in bone cancer research. It is responsible for essential processes within cells such as protein transport and processing, as well as sorting, which help ensure homeostasis in cells. Dysregulation of Golgi-mediated membrane trafficking can have severe repercussions for bone health and the risk of cancer development. Changes to membrane trafficking within the Golgi apparatus can disrupt cell functionality in sensitive tissues such as bone, potentially leading to cancer formation and progression. Understanding the relationship between Golgi apparatus dysfunction and bone cancer pathogenesis is crucial to identifying potential therapeutic targets and devising new treatment strategies to combat this devastating illness. Further research in this area can deepen our knowledge of its intricate mechanisms, leading to improved diagnostic and therapeutic approaches in the future. The Golgi apparatus plays an essential role in cell biology processes, such as transporting, processing, and sorting proteins and lipids. Thus, its significance in cell science research has attracted widespread notice. Previous studies have highlighted the central role of the Golgi apparatus in many diseases. Through an examination of their pathophysiology, it has been seen that most genes associated with such illnesses play an integral role in membrane trafficking processes. Notably, tissues such as the nervous system, skin, bone, cartilage, and skeletal muscle show particular sensitivity to irregularities in membrane trafficking. At this point, there can be little doubt that broadening our foundational knowledge of Golgi-mediated membrane trafficking will provide key insights into genetic diseases. Furthermore, ongoing investigations of such illnesses will increase our comprehension of its involvement in disease processes.
